# On the Use of Chloroform in Midwifery

**Published:** 1850-01

**Authors:** Geo. N. Burwell

**Affiliations:** Buffalo


### BUFFALO MEDICAL JOURNAL

AND

### MONTHLY REVIEW.

VOL. 5. JANUARY, 1850. NO. 8.

### ORIGINAL COMMUNICATIONS.

ART. I.—*On the use of Chloroform in Midwifery.* By GEO. N. BURWELL, M. D.

In the number of this journal for June last, I indicated my intention of giving such views and reflections as I had formed from my own experience, respecting the use of Chloroform in Midwifery. I propose now to carry out that desire.

My remarks will be founded upon the observation of sixty-two cases, viz: the series of fifteen cases given, in some detail, in this journal for November, 1848; the series of twenty-three cases, given in like manner, in the number for June last; and twenty-four cases in which I have since administered the remedy.

I shall give this analysis of cases with that degree of minuteness necessary to their full appreciation, even at the risk of being considered unnecessarily exact. On a subject of such universal importance as the use of Anæsthetics in Midwifery, and upon which there is such a contrariety of opinion, a loose, general summary of cases would be alike uninstructive and unsatisfactory to those wishing to examine the subject with care and precision.

They may be divided, for review and examination of the results obtained, into four classes, as follows: 1st, six forceps cases, in which chloroform was given during the use of the instruments; 2d, one case of turning; 3d, thirteen cases of unassisted labour, in which there was complete anæsthesia at the time of the birth of the child; and 4th, forty-two cases in which the remedy had been so administered that while (with three exceptions) it afforded relief, entire anæsthesia was not at all, or never more than momentarily produced.

*First*—Of the forceps cases. There is a great similarity in *five* of these cases. The head in each one, had reached the inferior strait, and the application of the instruments was made with great facility, and the delivery rapidly accomplished. The effect of the Chloroform was precisely alike in all five. There was no remembrance, in any one, of the birth of the child; neither were there any cries of distress. The muscular system was without contraction, except a moderate use of the voluntary muscles (and probably also of the uterine muscle) with each attempt at extraction. Twice I applied the forceps while the women were anæsthetic, but prefer, and recommend that they be introduced and locked before giving the chloroform. All the women did well. Three of the children were still-born (see paragraph on still-born children, page 454,); the other two did well.

In the sixth case, chloroform had been given between three and four hours before the application of the instruments. Ergot had also been given. The head had ceased to advance after it had got well into the cavity of the pelvis. It was the first child. The forceps were used to bring the head down against the perineum; this being accomplished, the instruments were removed and the completion of the labour left to nature. This was done on account of the rigidity of the perineum, and a fear of laceration, if the delivery was hurried by the farther use of the instruments. Chloroform was continued all this time, just as it had been given before the use of the forceps; that is, to benumb the pains, but not to cause full anæsthesia. Both mother and child did well.

*Second*—Of the Case of Turning. This occurred since my last publication. The woman was a patient of Dr. Dayton, of Black Rock. She lived some miles distant from that village. Doct. Dayton was sent for in the evening of August 10th, and finding the labor progressing slowly, laid down. He was hastily summoned to his patient about 4½ o'clock in the morning of August 11. He found that the waters had escaped while she was on the close-stool, and the child's right hand was protruding into the world. The patient finding that something was wrong, and thinking death to herself certain, in spite of the Doctor's assurances of her entire safety, gave herself up to the most immoderate weeping, and other evidences of excessive grief. Doct. Dayton attempted to turn the child and deliver, but such was her restlessness and violent outcries, that he desisted, and sent for me. It was four hours after the coming down of the hand before I reached the house. The woman had in a great measure got over her alarm, and was waiting very patiently. There had been no return of pain during all this time. The prolapsed member was much swollen; a spasmodic contraction of the fingers on my taking hold of the hand, proved the child to be living.

After having her properly placed, Dr. Dayton administered the chloroform slowly, but in full dose, so she soon became entirely anæsthetic, when the sponge was at once removed. Not over twelve or fifteen minutes were consumed in turning and delivering the child. It was accomplished with great ease, although the limbs were so folded upon themselves, that I had to reach into the very fundus of the womb before I could conveniently seize one limb. The child turned readily, and the arm gradually receded, but not entirely, until I had withdrawn the left foot (so the right hand and the left foot were at the same instant without the vulva), and soon after wards the child was delivered, living. The patient laid in the most quiet manner all this time; she did not utter a single cry or moan. Once she became for an instant a little restless, but three or four additional inspirations of the chloroform perfectly quieted her. There was no perceiveable contraction of the voluntary muscles during the delivery. The uterus contracted down well, and the after-birth was delivered in due time, and without hemorrhage. The patient did not wake from her sleep for some four or five minutes after the child was born. She recovered without a bad symptom.

It has been my lot a number of times to deliver by turning, but never before have I accomplished it, at full time, with one half the ease and rapidity I was enabled to in this case.

*Third*—Of the cases of total anæsthesia, not instrumental. Many persons associate the state of anæsthesia with a state of sleep; that is, a condition of muscular repose, with apparent insensibility to pain and to mental impressions. These conditions, although a common result, are not always to be obtained from the safe use of chloroform.

I propose to state the facts observed in these cases of total anæsthesia, (thirteen in number) under three heads, viz: 1, the effect produced upon the muscular system; 2, that upon the cries of pain; 3, that upon the mental faculties.

1—Of the muscular system. In no case were any of these patients so perfectly anæsthetic as to cause any diminution of the contractile power of the uterus. Twice I remarked an almost total loss of the action of the voluntary muscles, there being, at the time of the birth of the child, no action of those of the arms and legs, and only a slight contraction of those of the abdomen. The uterus, however, acted with full power; and it was interesting to notice with what force, in both cases, the children were thrown into the world. In two other cases there was a near approach to this state, but not such an entire loss of action. In one case, all the voluntary muscles acted much better under the chloroform than without it, and a labor (see case 8, first series) which promised to be tedious, was quickly terminated by the regular, powerful action of the accessory muscles. There was great restlessness in this case, which the chloroform controlled; and in this way hastened the termination of the labour.

Once I found a somewhat similar restlessness to remain uncontrolled by the chloroform, although given to complete anæsthesia of nearly an hour's duration (see case 13 second series). So far from quieting this irregular muscular action, it became more and more marked, as the pains increased in severity, toward the conclusion of the labour.

In the remaining seven of the thirteen cases, the state of total anæsthesia did not interfere with the full action of the voluntary muscles. They contracted as quickly, and with the same power, as in ordinary cases of labour without the use of chloroform.

2—Of the effect of chloroform upon the exclamations of pain. In six of the thirteen cases, the children were born without any cries or other manifestations of pain, viz: in the four cases in which the contractile power of the muscles was lost or diminished; in the case in which these muscles acted so much more vigorously; and in one case in which there was the normal action of these muscles.

In five cases there were moderate complaints, such as crying out, or moaning at each recurrence of pain, with an increase of these expressions at the moment of the birth of the child. In all these (and in one particularly so) the outcries were much diminished in comparison to what they had been before the inhalations.

In two cases the cries were as loud, or nearly so, as before the use of chloroform. It is only because I have confidence that the women spoke the truth when they afterwards declared their entire insensibility to the delivery of the children, that I place the cases under the head of total anæsthesia.

3—Of the condition of the mental faculties. In six of the thirteen cases, there were no mental manifestations. They laid perfectly quiet in the interval of pain; made no answers to questions; nor obeyed any directions about change of position. Two of them were those just referred to as making such loud outcries during pain; two were of those who made only moderate complaints, and two of those who did not utter any cries of pain.

In three cases the only manifestation of intelligence, was a condition approaching to dreaming, with muttering, viz: one repeatedly spoke her husband's name, as if calling to him; one muttered something about her child being likely to be injured; and in the third, the subject was of an agreeable nature, her exclamations being, "Oh! it is pretty! it is pretty!" None of these cases uttered any cries of pain.

The remaining *four* cases were apparently so sensible, responded so correctly to questions, and followed any directions given, so promptly, that only their own declarations of insensibility to the birth of the children, would be able to convince any one present, that such was really the case.

Two of the cases I have already published (see cases 6 and 8, first series). With the first of these was combined some muttering delirium, which I have learned very generally to associate with insensibility. In the other, instead of delirium, there was a tendency to weeping and such like hysterical symptoms, also generally indicative of insensibility.

The other two cases have lately occurred to me; and as it is in similar cases, as it seems to me, that we are most likely to push the remedy beyond the bounds of prudence, I think them of sufficient interest to be reported at some length.

In the first one, I commenced the use of chloroform one hour before delivery; the os-uteri was nearly to its full dilatation; first child; the woman of a delicate constitution; highly nervous; making loud complaints of the severity of her pain; no feverish symptoms; pulse 80, regular; had been unwell for two days with a threatened dysentery. It was from twelve to fifteen hours since labour pains had commenced. She first breathed of it for a pain or two, as an experiment, and found that it relieved her; and afterwards asked or made signs for it, by raising her hand, at the commencement of every pain, until delivery. I do not remember that she once failed to do this. When given to her, she would breathe of it with the greatest avidity, pressing the sponge to her mouth and nose as closely as we would allow her. Frequently she wanted it (the sponge) made stronger; that is, more perfectly saturated with the chloroform; once she spitefully threw it from her from disappointment, on its being handed to her without a fresh wetting. A number of times during the hour I asked her to reach me her hand for the purpose of noticing the condition of her pulse. She always complied at once. She would as readily comply when told to change her position, or to raise her head to fix the pillows under it. She would frequently say something about the severity of her pain, and how she suffered, and always made more or less complaint with each return, but very moderately so in comparison to what it had been before breathing chloroform. Only four or five pains before the birth of the child, she asked me if she could not be put to sleep, and made quite insensible to pain, and desired that it should be done. I declined doing so, for, judging from the quantity of chloroform she had taken, I thought it probable that she would not recollect much about it afterwards.

She was all this time apparently entirely sensible; there was no delirium, nor any hysterical symptoms. There was no diminution of the force and frequency of her pains, or of the action of the accessory muscles. Indeed, I felt unusual interest to know whether she was really sensible enough to recollect afterwards all that had occurred.

She did not lie in a calm, quiet state after the birth of the child; but then, for the first time, shewed signs of mental aberration, by a disposition to loquaciousness, by asking the same question over a number of times' and by making remarks foreign to her condition. She still appeared to understand well enough, for a moment or so, what was said to her, and obeyed directions as well as before. Her movements were quickened; her mind was unusually active; her general expression of countenance was that of excitement and alarm; there was for a while frequent and unusually deep gaping; and the orbicularis palpebraris muscle would also frequently contract for a few seconds at a time, giving her eyes a very wild and unnatural appearance. The pupils were unaffected; the respiration was not affected except in this disposition to take deep inspirations; her pulse had risen before the conclusion of the labor, to 120, and now kept at that number, and perfectly regular. After lying in this state of excitement some twenty or twenty-five minutes, she suddenly settled into a state of perfect repose, and apparent drowsiness, and soon afterwards came to her full recollection.

She has since, a number of times, remarked to me that she has often tried to recollect when the child was born, but could not; nor can she recollect anything after the third or fourth inhalation, an hour before delivery, to nearly half an hour after this event.

She recovered well from the dysentery, being fully convalescent on the fifth day.

The other case was one in which I commenced the administration of chloroform before the full dilatation of the os-uteri. I kept it up pretty fully for half an hour. She said it relieved her, but did not call for it, nor shew anything of that avidity for it, seen in the case just related. Finding her progress rather slow, I left her for half an hour, and, of course, discontinued the use of the chloroform, for I never allow it to be given in my absence. On my return, I was told that she had asked quite earnestly for it. I commenced its use anew, and gave it pretty steadily for one and a half hours, until the completion of the labor, only once discontinuing it for eight or ten minutes, as she expressed a wish to get up and walk the room, thinking she would be easier by so doing; thus shewing that although partially under the influence of chloroform, she was fully sensible to her pain. But her pains were increased by this, and she soon got on to the bed again, and called for more chloroform. This was about all she said, unless asked, that shewed her appreciation of the relief obtained from the remedy. It was evident to all that her cries were much lessened by the use of the chloroform, yet she cried out, about her back, almost every pain, generally placing her hand there, the moment it came on. When asked, she said the chloroform relieved the pain, and prevented it from being continuous. She did not shew the least sign of aberration of mind, nor of hysterical symptoms. Her pulse remained entirely unaffected. She frequently dropped into a sleep, the moment the pains went off. The child was born without her being at all sensible of it; and she afterwards declared that she remembered nothing of the last hour and a half, except of getting up and walking the room.

Her condition after the birth of the child afforded a marked contrast to the last case. She laid so very still, although awake, and said so little, that the attendants noticed it as something unusual, and asked if it was the effect of the chloroform. This state continued for an hour, during which time she scarcely spoke, unless spoken to, or appeared to take much notice of anything; yet she answered all questions, at once, and correctly.

This was another case in which I found it impossible to be perfectly satisfied, before the birth of the child, of the actual state of my patient. She would complain so constantly of her pain, apply her hand so promptly to her back, and obey so quickly, any directions given her, that I felt I could not safely make any predictions what would be the extent of her intelligence and memory.

These cases seem to me to be very important, from the difficulty and uncertainty in judging correctly of the actual condition of the patient, and, of course, of the propriety of giving more chloroform. If accidents are to occur in the use of chloroform in midwifery, it will be, most likely, in such cases as these, from urging the remedy under a false impression of the actual sensibility to pain, on the part of the patient.

*Fourth*—I come now to the consideration of the *forty-two* cases in which chloroform was given, but not to the extent of the abolition of the senses.

I commenced the use of chloroform, influenced by the recommendation of Prof. Simpson, that it was better and safer to give it in full doses, and to bring the patient quickly under its influence, than to give it in small doses; and for this reason, I gave it in my first cases in such a dose, and to that extent. The result was, that (with one exception) they were all soon anæsthetic. But on the 19th of April, 1848, a case occurred to me (case 9, first series) in which I gave it nearly every pain, for three-fourths of an hour, without causing more than a momentary loss of consciousness, and yet with great relief to pain. This demonstrated to me, so clearly, the possibility of giving decided relief without affecting the intelligence of the patient, and seemed so preferable to the course I had adopted, that I determined thereafter, except for especial reasons, to seek that medium, of which this case afforded so good an example. This is why such a large proportion of my cases come under this head; and it would have been still larger, had I not, in eight cases, produced entire anæsthesia, without, at the time, intending it; from the difficulty there is, in every case, in reaching that medium effect.

Thus, while some have been unintentionally made quite anæsthetic, I have, as undoubtedly, in other cases, administered it less freely than I might have done, to have given even greater relief, and yet have left the mind perfectly clear.

Three of the forty-two cases did not obtain much relief from pain, although I thought I gave the remedy a fair trial; three or four others appeared to derive but moderate relief, while, in all the remainder, the relief obtained was very decided; the patients often giving expression to their feelings of comfort with the utmost promptness and earnestness. In quite a number of the cases, the children were born without a single cry of pain, so I have thought them insensible, until assured to the contrary. The large majority of them, however, made more or less outcry, with every pain; in many it was quite loud; in others, very moderate.

I have seen it two or three times cause an exhilaration of feeling and spirits. They would become cheerful, laughing and talkative, (but not at all incoherent) where, before, all had been gloom and complaint.

But the rule, according to my observation in these cases, is, that it does not alter the prevailing turn of the patient's mind and feelings, farther than that she does not complain so much of pain. If she was complaining before, she still continues to do so, but in a more moderate, subdued way; and whether she was disposed to be lively and talkative, or dull and apathetic, saying little or nothing, she remains so still; although declaring her relief, and in many cases constantly begging for more of the remedy.

It cannot, of course, be expected that the chloroform would have much influence over the muscular system, when so given as not materially to affect the mind. I have only to report under this head, that in one case, (see case 21, second series) almost every time it was inhaled, it had the effect of lengthening the intervals between the pains, by stopping all uterine contraction; and I think I could have kept off the pains with it, perhaps, for an entire day. The patient had not slept of any consequence for nine days, on account of continual pain, and an inability, therefrom, to lie down. The result was, that the moment she got any relief, she fell into a snoring, apoplectic kind of sleep. Of course, I used it very sparingly, after noticing this effect. This has been the only case I have seen of any decided, certain retardation of labour from the use of chloroform.

In another case, I think, without doubt, it hastened the termination of a labour, by "taking away," as the patient expressed it, a sharp pain in her bowels, which prevented her from bearing down. This was the second, of the only two cases I have seen, where I have felt sure of its being the means of shortening a labour.

In six or eight of these forty-two cases, I have given it to the extent of causing a momentary delirium, similar to that noticed under the head of full anæsthesia. By withholding the sponge for a pain or two, this would pass off, and the patient again become fully sensible.

One patient who declared herself to have been fully sensible all the time, said that the two hours I had been with her, administering the remedy, did not seem to her over half an hour. She was not probably aware how much she slept between the pains.

It has been a common thing to see them fall into a quiet sleep during the intervals of pain, but to be fully awakened on its first recurrence. Often, the first intimation I have had that pain was coming on, was a sudden start from sleep, and a call for more chloroform.

In one case I thought I had tried it thoroughly, and would have to report it as a case of failure. Some ten or fifteen minutes after I had laid the remedy aside, (the pains following each other very rapidly) I was surprised to hear her call for it, and insist upon having it. She continued to call for it until the end of the labour; and I have never had one express more satisfaction with it. She said that at first, although it relieved her very much, it made her head feel so dizzy and queer, she was afraid of it; but finding all this pass off, soon after its discontinuance, she concluded to take it again.

In another case, I gave it in like manner, and laid it aside because she did not shew that appreciation of it I like to see. After the conclusion of the labour, she said, that at first, she thought she would take it, but became afraid of it, although sensible of relief while she was breathing of it.

*Five* of the forty-two cases were forceps cases, in which chloroform had been given before the application of the instruments, but not at the time. The reasons for this discontinuance, were in *one* case, that a pain came on immediately on the application of the instruments, and advantage was taken of it to deliver, without waiting to give chloroform.

The *second* was a case in which difficulty was apprehended on account of a face presentation, where the instruments had to be applied across the face (case 10, second series).

The *third* case was one of those in which its previous trial had not given much relief.

The *fourth* and *fifth* cases required the use of the instruments on account of the large size of the children, especially of the head. The delivery was slow, and required a great deal more force than I was willing to exert with my patients in an insensible condition. One of these cases is published in the June number of this Journal, (case 23) and is a good example of the other one.

The remaining thirty-seven cases were all of natural labor, with one exception; a case of twins (premature). Some were rapid cases; other tedious; and the chloroform was accordingly administered from a few minutes only, up to a continuous period of five and a quarter hours.

I propose next to notice my observations in a number of interesting particulars, without which the subject would be quite incomplete.

1. The first and most common effect experienced on breathing chloroform, is an universal thrilling or whirring sensation, which goes through the whole system, even to the ends of the fingers and toes. Many have said that they have felt it go right to the back and numb the pain there. Besides this particular feeling, it produces, in some, a dizziness, a kind of faint, sickening sensation that is particulary disagreeable; noises in the ears are frequently experienced, and even light before the eyes (one patient only has said she saw balls of fire). These sensations are most generally the effect of the first inhalations only, and pass off as soon as the system gets a little accustomed to the remedy. A large majority of those who breathe it, however, do not experience these disagreeable sensations, and some, even, find it agreeable and exhilarating.

2. I think there is no doubt that the effect of the remedy is cumulative for a short time; not over half a minute, however, if given in the small doses in which I generally administer it. Having reached this point of greatest relief, it will be from one to two minutes before the effect passes off. Much will depend as to the duration of its effect on the size of the dose, and the length of time it has been inhaled.

3. I have never but once happened to smell it in a patient's breath, after she had stopped its inhalation. This was in the case in which its administration had been almost continuous for over five hours. Here, happening to lean over her to hear some remark, an hour after delivery, I found her breath to smell quite strongly of the chloroform. She did well in every respect. Does not this case tend to show that it is not in saturating the system with chloroform that there is danger, but more probably in the production of a sudden effect, either by too large doses, or carelessness in neglecting to secure the due admission of air. I have seen, in a few cases, the difference between breathing it without air or with air. Some of my patients have shown such avidity for it, that they have pressed the sponge so close to their faces as to exclude air, and I have noticed how much quicker they showed the effects of it, than when the sponge was held off a short distance, and a plenty of air admitted. It did not take half the time, nor half the number of inspirations to produce the desired effect.

4. I have met with one person who could not inhale it. She was anxious to do so, and tried faithfully, but absolutely could not bear it about her face. It "stifled" her, as she termed it—that is, stopped all power of inspiration.

5. When I commenced the use of chloroform, I laid down to myself the rule not to administer it if the pulse was above the natural beat, and I have generally been governed by it. The exceptions are four, as follows: Once when it was permanently at 96 to 100, but uncomplicated with fever; once when it was 110, once 130, once 120, with fever, (see cases 7, 8, 19, and 21, second series). All these were tedious cases, and all had been previously bled; three during labour, and one thirty six hours after labour. In one case only did the blood show more than a light covering of fibrin. The chloroform acted as well as though no such complication existed. In all, the feverish symptoms subsided spontaneously in two or three days after delivery.

Four times I have seen the pulse rise during its administration, or immediately afterwards, from 80 or 90 to 120, and over. One had puerperal fever, of which there was no sign before delivery (see case 2, second series). The other had dysentery, but which had already commenced before the labour, (see case on page 445 of this number). The first required free depletion, and the blood taken (as was also that taken during the labour, and before any rise in the pulse,) was highly buffed and cupped. The second case did not require depletion, but was promptly relieved by other means. In the other two cases, the rise was in consequence of hemorrhage, it not having risen until the occurrence of the accident. (See case 7, second series for one case; the other case occurred since that publication, and has not been reported.)

With these exceptions, I have never seen any permanent variation in the pulse which I could, with certainty, refer to the chloroform. The more general effect, if it is altered at all, is a rise of ten or fifteen beats; in but two or three instances, at most, have I noticed a fall, similar in extent.

6. I have never given it so as to produce stertorous breathing, aor any other marked effect upon the respiration. I have been agreeably disappointed in its liability to excite cough, never having seen any such effect, except occasionally, upon the first inhalations; and such disposition has invariably passed off after a few inspirations.

I have had one patient (a case of total anæsthesia,) complain for a few hours after getting sensible, of an oppression about the chest—a kind of inability to expand the lungs.

7. I had anticipated, from what I had heard, that vomiting was a frequent result of the inhalation of chloroform. It has not been so in my experience. There has not, in these cases, been over the average of sick stomach and vomiting during the progress of the labour.

8. I have given it in three cases where decided headache existed at the time. Two said that it relieved them, and one, that it had no effect upon the headache. I had previously bled one of those put down as relieved, on account of the excruciating character of the headache, (far more severe to her than the labour pains), with turns of blindness; and as I greatly feared, an attack of convulsions, (see case 20, second series). The venesection gave great relief of itself; the chloroform afterwards gave still further relief.

I have never seen the remedy cause any serious headache. A number of times I have had complaints of dizziness and other disagreeable sensations about the head. One patient said it made her feel as she did once when a child, from getting intoxicating liquor, incautiously left within her reach; another said it made her feel precisely as she once did when magnetized.

Once I had a patient complain of getting faint. I was administering the chloroform to her while she was sitting up.

9. While I do not think that chloroform either increases or lessens the liability to hemorrhage, I have to state that I have had two bad cases of this accident follow its use. One of the cases is given in the June number, (case 7). The other was a woman who always flooded badly after labour. I was not aware of the fact, never before having attended her. She had been breathing the chloroform for three-fourths of an hour before the birth of her child. It was one of the most severe cases of hemorrhage I have ever met with.

I gave it, in August last, to a patient also subject to hemorrhage. It was her third confinement. I administered pretty freely of an infusion of ergot in the intervals of the pains and the giving of the chloroform; and succeeded both in preventing the flooding and in easing her pains.

10. I have had one case of puerperal fever follow its use. See case fully reported (case 2, second series).

11. A few have complained of burning, and of slight excoriation of their lips from its use.

12. I have not met with what I could consider an unequivocal case of its aiding in the dilatation of the os-uteri or perineum, or of promoting secretion from the vagina. I have seen cases where the process of dilatation has been unexpectedly rapid, but I have seen just as many similar cases in which chloroform has not been given. Again, in a number of cases in which I have administered it the longest, and the relief obtained had been perfectly satisfactory, the labour has been delayed beyond what I had hoped, by a rigid os-uteri and perineum; and where I have looked for and anxiously expected such a result from its use.

13. I do not think it disposes to asphyxia of the child, or to cause it to be still-born. I have seen two children partially asphyxiated. Both were from primaparæ; one had the cord twice tightly around its neck; the other was after a tedious and severe labor, in which I had used chloroform three and one-half hours. The child of the woman in whose breath I smelt the medicine one hour after the conclusion of the labour, cried out lustily the instant it was born.

I have delivered seven children, still-born, from six mothers, to whom chloroform had been given. Two were twins, at four or four and a half months; two were hydrocephalic children, and both with spina-bifida; one was putrid, and had been dead at least two weeks before the labour; one died from prolapsus of the cord; and one was a case in which I used the forceps, and was undoubtedly dead before the giving of the chloroform, or the application of the instruments.

14. I have not observed it to have any influence over the after-pains. I have seen them very severe in two or three instances after its use; also some cases in which they were much lighter than they had usually been. I was asked by one woman, who had them severe, and who had taken it satisfactorily for the labour pains, to give it to her for the after pains, but I declined, never having given it for this purpose.

15. I have not experienced any thing even approaching to muscular spasms from its use, nor, with the exception of the case given on page 445, of any nervousness or aberation of mind that lasted five minutes after the inhalations were discontinued; nor have I had a case of death from its use.

The more particular objects for which chloroform is given in Obstetrics are four. 1st, For the relief of pain, whether the labor be natural or assisted; 2d, for the promotion of the dilatation of the os-uteri and perineum; 3d, to control puerperal convulsions; and 4th, to facilitate turning.

The *first* of these objects is almost the only one I have in view, in administering the remedy. I have already expressed my doubt, as to its power to promote the dilatation of the soft parts. I have seen but one case of puerperal convulsions since I commenced the use of chloroform, and that was so soon and so well controlled by a large bleeding, that I did not give chloroform; and, therefore, cannot express an opinion upon this point, derived from my own experience.

In the relation of the case of turning, I remarked the ease with which it was accomplished. But a single case cannot determine any thing. It has only led me to look upon the remedy very favorably in similar cases. The relief from pain, then, being, according to my observations, the great indication for its use, and it being ascertained by trial, that some will continue to moan, and cry out, and give other indications of pain, and of apparent intelligence, even when they afterwards declare themselves to have been, not only totally insensible to pain, but unknowing to every thing that had been said or done, even to the birth of the child, it becomes important to ascertain if there be any symptoms in these cases, by which we may know whether or not the patient is insensible, so as not to urge the remedy in larger and larger doses, under the false impression that she continues to suffer.

The following rules which my own experience has taught me to lay down for my guidance, in judging when anæsthesia has been carried far enough, I would recommend to the observation of others for verification, and if found to be reliable, to be carefully followed in practice:

*First,* When there is delirium, as shown by incoherent muttering, or talking about irrelevant subjects, however slight the reference may be to them; or when there are hysterical symptoms, as sobbing, or a disposition to cry, the patient will afterwards be found to have been insensible while these symptoms have lasted. There has not been an instance to the contrary in my experience. The administration of the chloroform should be then immediately discontinued, unless the object be to keep the patient in this state, when the amount should be lessened.

*Second,* There are many who will declare at once the relief they obtain, and demand the remedy for almost every pain throughout the labour. In such cases, their declarations afford sufficient indications of relief. Others will not tell of their relief, but it will be noticed that they do not cry out with the earnestness and sharpness they did before. Their expressions of pain become more like moaning, and they give utterance to them only at the time the pains are on. If they are thus changed in a decided manner, anæsthesia has generally been carried far enough.

*Third,* When they fall into a sleep immediately a pain leaves them, and sleep quietly on until suddenly roused by a renewal of pain, I consider them to be sufficiently anæsthetic, although they may make considerable outcries at the time, or even speak with perfect intelligence. I do not, therefore, make any increase in the dose, but rather diminish it as soon as the effect is observable.

*Fourth,* If I notice any positive diminution, ever so slight, in the action of the muscular system, I consider the patient sufficiently anæsthetic. But it ought also to be remembered that the reverse of this does not hold true; and that strong muscular action is not incompatible with total anæsthesia. This renders the condition of the muscular system an uncertain means of judging of the real state of the patient. We have seen in the cases of total anæsthesia, that in nine of thirteen cases, it had no effect to weaken its action. We have, also, seen in one case, that irregular muscular action was instantaneously controlled; while in another case, no such effect was produced.

It is to a want of knowledge of this fact, or to carelessness in reference to it, that I refer, in part, some of the fatal surgical cases from the use of chloroform. The subject, on the commencement of, or during an operation, has been restless, stretched or thrown out his limbs, or made expressions of pain; and sensibility being supposed to exist, more chloroform has been administered, and he has thus gotten an over dose. Might I not have had the same accident happen to myself, if, upon a similar supposition, I had urged the use of the remedy in some of the cases I have narrated? I think very possible; and I would suggest particular caution against endeavoring to control muscular restlessness; or, because the patient utter cries of pain, think that all must be stilled, or the use of the remedy will not be sufficiently brilliant.

*Fifth,* The amount of chloroform you find yourself giving, will often afford a good criterion by which to judge of the amount of relief the patient will afterwards express. Used without water, and in the cautious way I do, half an ounce an hour will almost always be found to render a patient entirely insensible, as to any after remembrance of the labour. The woman who did not come clearly out from its effects for half an hour after the conclusion of the labour, used five drachms the hour previous to the birth of the child. I would, therefore, seldom give over this quantity an hour, however great, apparently, their pains might be. From two to three drachms an hour will generally be found to be all-sufficient to keep them tolerably relieved, if the pains are not very rapid and sharp. As I seldom give it with the intention of producing full anæsthesia, I rarely exceed that quantity an hour.

I place the contra-indications to the use of chloroform under the following heads. I have not invariably been governed by them, but consider them as general rules, additional to those already given, for my guidance in the use of the remedy.

*First,* The existence of a rapid pulse, and feverish symptoms have, with four or five exceptions, deterred me from its administration. I would also recommend its discontinuance if any marked acceleration or diminution of the pulse should be observed. I once continued its use after a rise of forty beats a minute, but would not do so again. (See case on page 445.) Any marked irregularity of the pulse, or stethoscopic signs of diseased heart would be positive contra-indications.

*Second*, I have, in four or five instances, where the patient has been flushed and hearty, delayed giving it until after venesection; looking upon plethora of the circulating system as contra-indicating its use. I would not be understood, by this, that I have ever bled under the sole idea of making the administration of chloroform more safe. I have always, on the contrary, let the employment of venesection depend upon my judgment whether the patient would not do better for it, entirely irrespective of other considerations; her own comfort and safety being the objects sought. Having secured this, if I thought she could be made still more comfortable by the use of chloroform, I have not hesitated to administer it.

*Third*, Diseased lungs have always been deemed a contra-indication. "Weak lungs," as they are called, have always, in my practice, forbid its employment. If I have known them to be subject to a chronic cough, I have always kept silent about its use. How far subsequent experience may alter or vary this rule, I will not venture to predict. Cases are on record of its use, with impunity, where a bad cold existed at the time; but I would exercise caution in such cases, and for the present, at least, look upon such a condition of the lungs as contra-indicating its employment.

*Fourth*, If I find, on trying it, that my patient does not breathe it readily and freely, nor make any expressions of relief, I generally stop its farther administration. They often will call for it after a while, and wish its continuance, when at first they would say nothing in its favor. Only three or four times have they neglected to do so.

*Fifth*, A few times I have seen reason, in the course of the labour, and after I had commenced the use of chloroform, to change my prognosis from a favorable to an unfavorable one. This fact, until now, has led me to discontinue its use, and always, as the result proved, needlessly so. I did not wish to hazard the possibility of the unfavorable result being laid to the chloroform, which would very naturally be the case, it being a new remedy. So a prospect of difficulty and danger has been, with me, a contra-indication to its farther use.

*Sixth*, Another contra-indication is afforded in those cases of retaradation of labour from a large sized head, or from locked-head. We know that in some cases, at least, chloroform will lessen the full contractile force of the voluntary muscles. Now, before proceeding to the use of instruments, or administering ergot, I would discontinue the chloroform to see if the patient might not make better progress without it. If not, its use might be resumed, combined with such other remedies as the case might demand.

*Seventh,* We sometimes see patients whose last pains are so very rapid and forcible that they hardly have time to get breath. The whole face becomes greatly congested, and without doubt the brain is similarly affected. I have, in such cases, great scruples about giving much chloroform, especially for the first time; and generally consider such a state of things to contra-indicate its use.

*Eighth,* Any objection to its use on the part of the patient, or by her husband, ought always to be a sufficient reason for not using it. It should never be urged upon any one. If the simple expression, on my part, that there is a remedy for the pain my patient is suffering, and that I consider it safe and prudent to try it, does not cause her to ask for it or about it, I never say any thing more, but let the labour go on without it.

*Of the mode of Administration and Dose.*—I have always used the sponge from which to give chloroform. It is a small piece, lightly stitched to the centre of a piece of oiled silk, some five or six inches square. The oiled silk prevents a too free evaporation and waste, while the patient is using it; and by being wrapped around the sponge, accomplishes the same object when she is not breathing from it. By this care in its use, I am better enabled to judge of the quantity I am actually giving to a patient, and thereby to introduce more precision into its use; and precision gives safety.

I took the precaution of cutting down the sponge to such a size, that it would hold just a fluid-drachm of chloroform when filled to saturation. By this means, an over-dose cannot incautiously be given. I very rarely fill the sponge, but generally have found it necessary only to about half fill it. If this quantity be given on the recurrence of every pain, and the patient breathe freely of it, she will almost inevitably soon become entirely anæsthetic.

The doses, as I give them, are, 1st, a fluid drachm, the full dose; 2d, thirty to forty drops, an ordinary or medium dose; and 3d, fifteen to twenty drops, a small dose. Whatever the dose, I manage so to hold it, as to have the sponge just off from the mouth, with the silk but lightly spread over the face, so the air can get free admission. Holding it in this manner, I instruct the patient to breathe freely and deeply, much as one breathes after some sudden exercise, until I see some indications that she has enough. As my object is to relieve pain, without affecting the intelligence, I prefer the moderate or small dose. If the pains recur rapidly, I use the small dose. I have already given the rules by which I am governed in judging when it is safe to give it, and how far to carry it. I am careful not to give over half an ounce an hour. I always prefer to give it just as the pain is coming on. The patient ought to be instructed to inform you of its earliest accession, and if the sponge be immediately applied to the mouth, she will generally get sufficiently under its influence before the pain gets to its heighth, to have it decidedly benumbed. If not given thus promptly, the opportunity may be lost of giving it at all; for such, often, will be the disposition to hold the breath while bearing down, and so sudden will be the inspirations, that it cannot be taken. So we ought to be ready to give it at a moment's warning; and if our patient is sensible enough to give us notice of the coming of a pain, and will breathe freely of it, every thing will be found to proceed to the satisfaction of all. But if she will not do this, or cannot control herself enough to hold still and breathe of it until she feels relief, the result becomes more uncertain, and a great deal will depend upon the experience and tact of the physician as to the amount of relief his patient will obtain. If he is careless, he will be very apt to give too mnch; if over-cautious, not enough; and in either case, he will put down his experience and authority as unfavorable to the use of chloroform in midwifery.

I always prefer to have the patient breathe of it principally through the mouth. It is breathed of freer and more easily. The sponge ought to be so held as not to touch the lips, or they will be apt to become excoriated from the contact of the chloroform. Some patients breathe of it with such avidity that they will, if allowed to, press the sponge so close to the mouth as to prevent the ingress of air. This ought not to be allowed. It might be dangerous if the dose happened to be a full one, as they are so much sooner and more deeply affected when they breathe of it in this way, than if plenty of air be admitted. I have noticed this fact frequently.

I am careful to have my patients lying when I give it. It is the best position. If they wish to get up after having breathed of it, I always discontinue it, and make them lie some five minutes or more before acceding to their wish.

I would urge the propriety, at first, of giving it slowly and in very small doses, so as to accustom the patient to its use. I do not, for the first few inspirations, ever give it with the expectation of relieving pain. But after they have tried it, and, as it were, got acquainted with it, I give larger doses, of which they breathe freely and without apprehension, and obtain the desired relief. I would suggest whether, as ordinarily given, the time of inhalation has not been too long and the dose too large.

Chloroform is a powerful remedy. I am glad that it is so. If it could be used in any dose with a certainty that after a longer or shorter time the patient would surely come from its influence, and unharmed in health, even if in some points it would be useful, there is no conceiving the injury that might be done with it in the hands of the evil minded. Therefore, although powerful, as it is shown to be harmless in so large a proportion of cases, let us not cast it aside, but endeavor to find that medium compatible alike with safety and relief.

Perhaps, hereafter, when opinions become more settled, it will be found that it can be safely given more plentifully than I have directed; and I think that this, very probably, will be the case, judging from the large doses in which many now administer it. But, for the present, I had rather err, if err I do, on the side of over-cautiousness and safety.

There is one thing in the use of the remedy I would be particularly glad to see. It is a greater uniformity in the manner, and more precision in the dose in which it is administered. In the great variety of modes and doses in which it is now given, we cannot judge so readily of the effects of the remedy, or know so well what symptoms may be attributable to it. All becomes confused. The experience of one physician does not go to correct or verify that of another, but, almost of necessity, is different. One will think that it retards labor; another sees no such effect; one will have accidents from it, another will have nothing but success, until amid such contradictory statements the profession lose confidence in the remedy and in themselves.

Let, then, a greater uniformity of practice be introduced, and a closer attention to the indications and contra-indications for its administration be observed, and I cannot doubt that the popularity of the remedy will increase, both with the profession and the public.

Buffalo, December 1, 1849.

